# The complete mitogenome of *Torodora canaliculata* (Yu & Wang, 2022) (Lepidoptera: Lecithoceridae) and its phylogenetic implications

**DOI:** 10.1080/23802359.2025.2468752

**Published:** 2025-02-20

**Authors:** Haotian Li, Sai Wang, Wenyu Liu, Huimin Yang, Xin Wang

**Affiliations:** College of Life Sciences, Liaocheng University, Liaocheng, China

**Keywords:** *Torodora canaliculata*, mitochondrial genome, phylogenetic inference, Gelechioidea

## Abstract

The complete mitochondrial genome sequence of *Torodora canaliculata* has been obtained based on the Illumina next-generation sequencing technology, which is currently the first reported mitogenome in the subfamil Torodorinae. The mitogenome is 15,590 bp in length, consisting of 13 PCGs, 22 tRNAs, 2 rRNA genes and one non-coding A + T rich control region. The phylogenetic tree constructed based on the maximum likelihood (ML) methods using the whole genome sequences does well support for the sister branch relationship between *T. canaliculata* and *Issikiopteryx taipingensis*. The complete mitogenome of *T. canaliculata* will provide useful genetic information for the evolutionary relationship of the Gelechioidea.

## Introduction

The *Torodora* Meyrick, 1894 is the most speciose genus in the subfamily Torodorinae (Lecithoceridae) with more than 200 known species, most of which are known from Oriental, Palearctic and Ethiopian regions. The incomplete taxonomy of this genus still leads to a large number of new species being continuously reported (Park and Koo 2022; Park et al. 2023). *Torodora canaliculata* Yu & Wang, 2022 was discovered in Yunnan, China in 2017 and is similar in morphology to *Torodora meyi* Park, 2008, but was described as a new species due to its differences in forewing and genitalia from *T. meyi* (Yu et al. 2020). The family Lecithoceridae (Lepidoptera, Gelechioidea) is a poorly known group of microlepidoptera, although it has a high species diversity (Park 2022). Moreover, there are fewer reports on the phylogenetic studies of the Lecithoceridae, and even its phylogenetic position in the Gelechioidea is still questionable (Kaila 2004; Wang and Li 2020). The mitochondrial genome has always been considered a powerful tool for constructing phylogenetic relationships. However, currently only one mitochondrial genome from the subfamily Lecithocerinae has been reported in the whole Lecithoceridae, while no mitochondrial genomes from the subfamily Torodorinae has been reported (Chen et al. 2022). Therefore, in this study, we characterized the complete mitochondrial genome of *T. canaliculata* and reconstructed the phylogenetic relationships of the Gelechioidea based on mitochondrial genes, providing some insights into the phylogenetic position of the Lecithoceridae.

## Materials and methods

**Figure 1.**
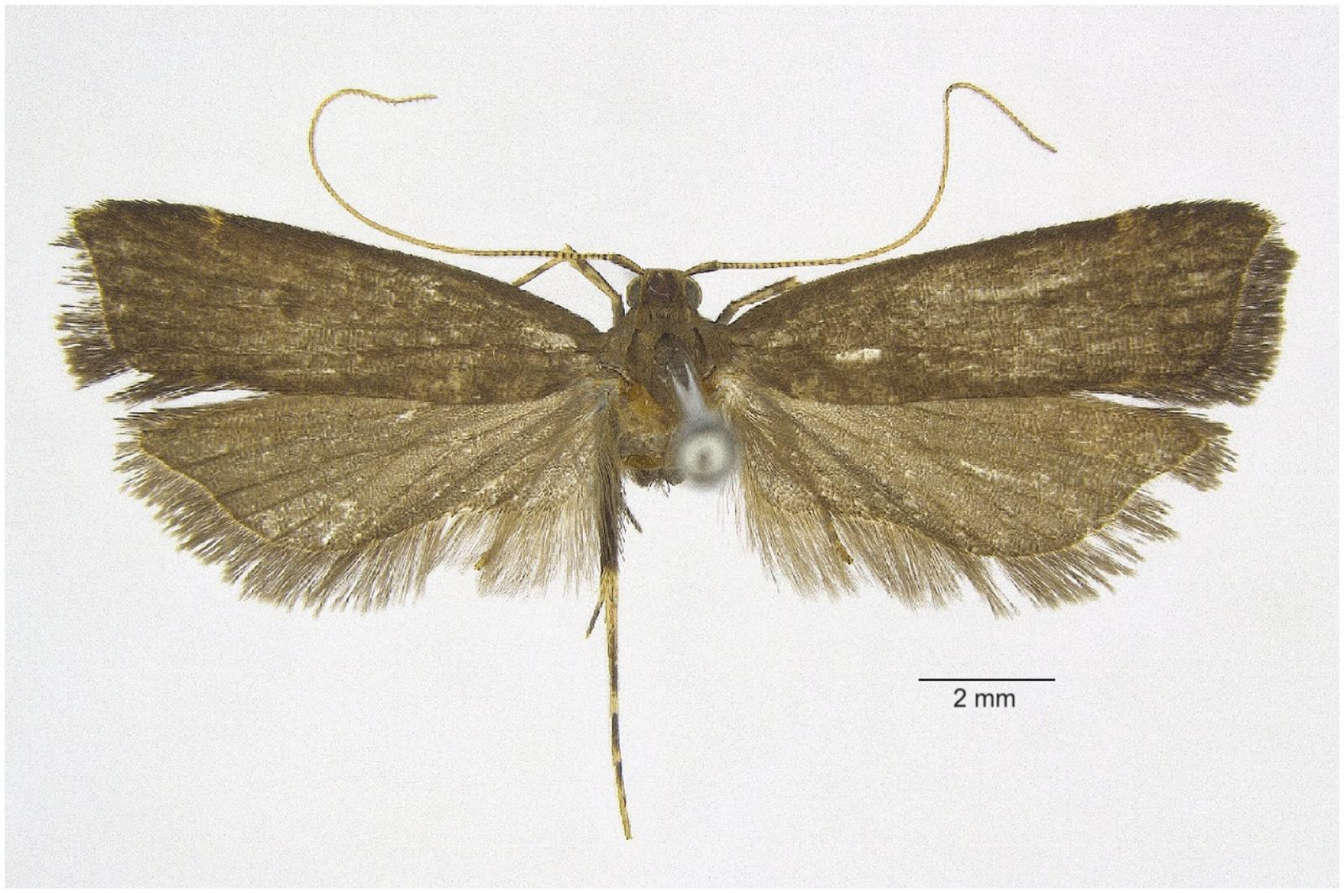
The morphological characteristics of *Torodora canaliculata*. The specimen in this photo was collected from Gongshan County, Yunnan, China (27°42′N, 98°16′E) The image was taken by the author (Haotian Li).

The specimens of *T. canaliculata* were collected in China using 250-W high-pressure mercury lamps on 11 Aug. 2022 at Mt. Banggunjian (24.39°N, 97.84°E), Mang City, Yunnan province by the license of the local Forestry and Grassland Administration. The morphological characteristics of *T. canaliculata* are shown in Figure 1. The specimens are deposited in the College of Life Sciences, Liaocheng University under the voucher number YUS024 (Dr. Shuai Yu, yushuai@lcu.edu.cn). The specimen has been identified morphologically by the grayish brown fringe of the forewing and the female genitalia by the rectangular signum of the corpus bursae to ensure the correctness of the species (Yu et al. 2022).

Total genomic DNA was extracted from legs using the Ezup Column Animal Genomic DNA Purification Kit (Sangon Biotech Co., Ltd., Shanghai, China). The DNA sample was paired-end sequenced by Illumina NovaSeq platform (Shanghai Personal Biotechnology Co., Ltd., China). A total of 24,868,092 reads (150-bp paired-end reads with a 300-bp insert library) were generated. The raw reads were quality assessed and filtered using FastQC v.0.11.9 (Andrews 2020) and NGS QC v2.3.3 (Patel and Jain, 2012) respectively. High-quality clean reads were used for the subsequent analysis based on Q20 (≥95%) and Q30 (≥95%). Using A5-miseq v20150522 (Coil et al. 2015) and SPAdesv3.9.0 (Bankevich et al. 2012) to reassemble high-quality reads into the mitochondrial whole genome. Annotations for the complete mitogenome sequence were generated using MITOS2 (Bernt et al. 2013). The mitogenome circle diagram was drawn using CGview visualization software (Stothard and Wishart 2005). In order to construct the phylogenetic tree of Gelechioidea, 21 available mitochondrial genomes from the Gelechioidea were downloaded from the GenBank database. The sequences of mitochondrial genome were aligned and examined using MEGA X software (Kumar et al. 2018). The three phylogenetic tree was performed maximum likelihood (ML) with the GTR+ G + I model using MEGA X software based on the all sequences of the entire genome, the full sequences of 13 protein-coding genes (PCGs), and the 1st and 2nd codon positions of the 13 PCGs sequences respectively. The bootstraps were obtained using a rapid bootstrapping algorithm with 1000 replicates.

## Results

**Figure 2.**
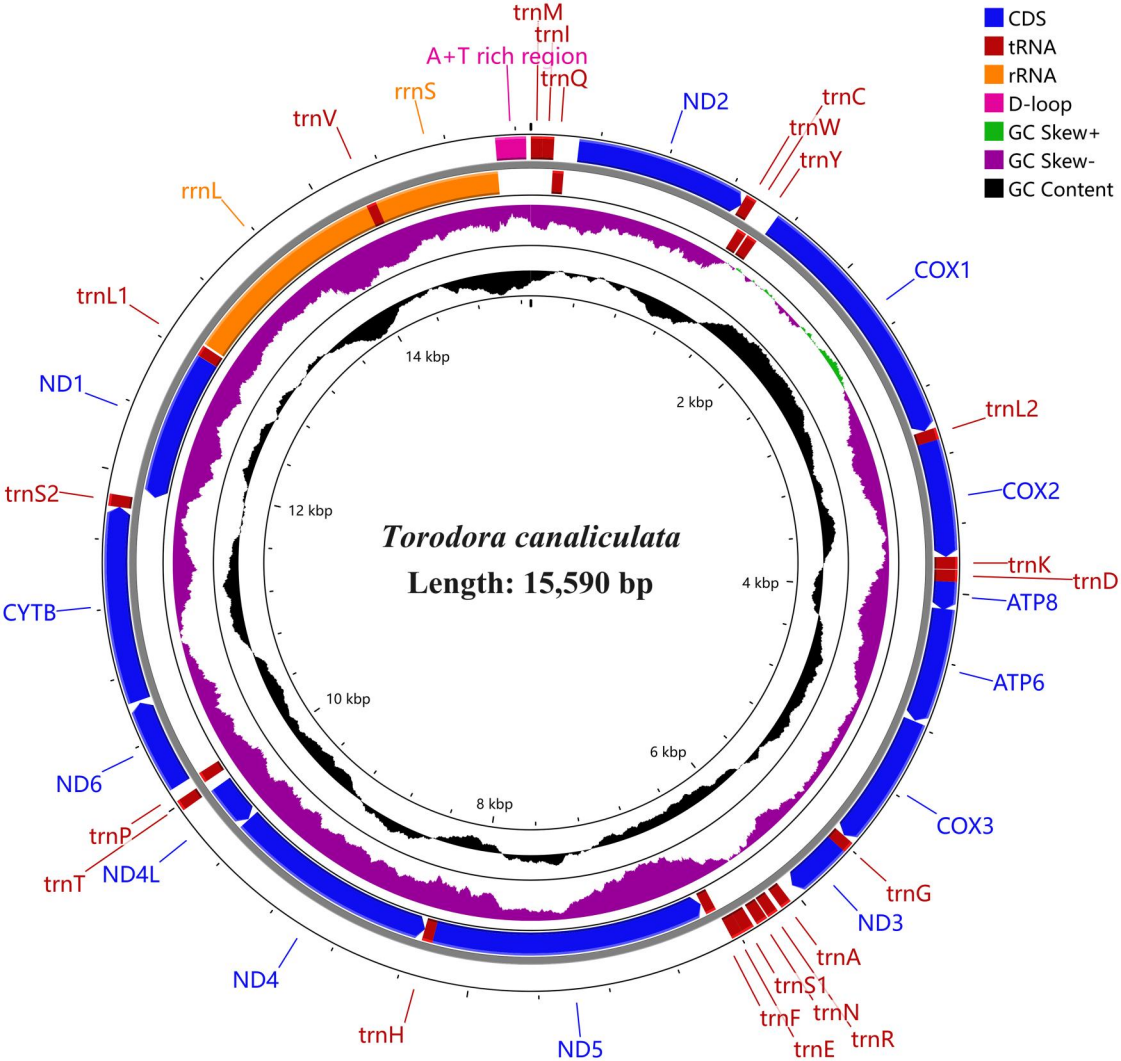
Complete mitochondrial genome map of *Torodora canaliculata*. From inside out: the first circle represents GC shew, the second circle represents GC content, and the third circle represents gene arrangement.

**Figure 3.**
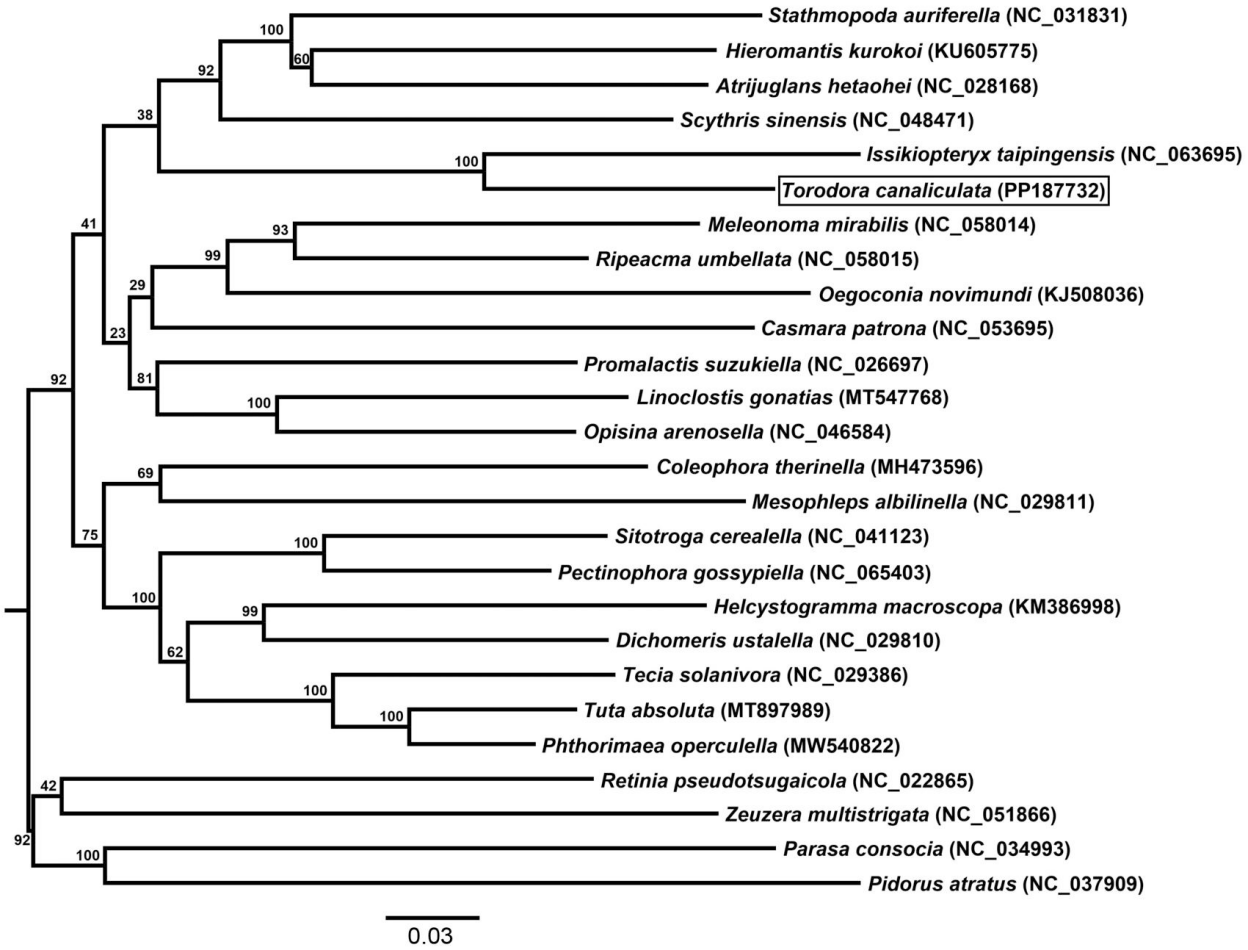
Phylogenetic relationship of Gelechioidea species based on whole mitochondrial genomes. The tree was constructed using the maximum likelihood (ML) with the GTR+ G + I model and 1000 bootstrap replicates. The bootstrap support circles are shown on each node. Scientific names and GenBank accession numbers are shown for each branch. The following sequences were used: KJ508036 (Timmermans et al. 2014), MT547768 (direct Submission), NC046584 (Meng et al. 2019), NC_031831 (Jeong et al. 2016), NC_026697 (Park et al. 2016), NC_058015 (direct Submission), NC_053695 (Jiang et al. 2021), MH473596 (Park et al. 2021), NC_029811 (Park et al. 2016b), NC_029810 (Park et al. 2016b), KM386998 (direct Submission), NC_065403 (Zhao et al. 2016), KU605775 (Park et al. 2016a), NC_028168 (direct Submission), NC_041123 (Yuan et al. 2019), MT897989 (direct Submission), MW540822 (direct Submission), NC_029386 (Viviana et al. 2016), NC_058014 (Yin and Yang 2021), NC_048471 (Park et al. 2020), NC063695 (Chen et al. 2022), PP187732 (In this study), NC_051866 (Li et al. 2018), NC_022865 (direct Submission), NC_034993 (direct Submission), and NC_037909 (direct Submission).

The complete mitogenome of *T. canaliculata* is deposited in GenBank under the accession number PP187732, and has a total length of 15, 287 bp. The overall base composition of the mitogenome is A (39.03%), T (41.33%), C (12.30%), and G (7.34%). It contains 37 genes without any other complex gene structures, including 13 PCGs, two ribosomal RNA (rRNA) genes, 22 transfer RNA (tRNA) genes, with 23 genes encoded on the heavy strand and the other 14 genes are encoded on the light strand (Figure 2). All PCGs initiate with ATN except for *COX1* which begans with CGA. Most PCGs use TAA as the stop codon; however, *COX1* and *COX2* used the partial codon T as the termination codon. The lengths of the 22 tRNA genes vary from 62 to 73 bp. The lengths of the *rrnS* and *rrnL* were 1335 and 782 bp, respectively. In addition, there is a non coding region enriched in A + T with a length of 178 bp. The mitogenome of *T. canaliculata* was assembled according to depth of the coverage (coverage of over 10× approximately) (Figure S1). In addition, overlap and noncoding bases in the mitogenome are also observed. We compared three phylogenetic trees, including a tree constructed based on whole genome sequences (Figure 3), a tree of the full sequences of 13 protein-coding genes (PCGs) (Figure S2a), and a tree of the 1st and 2nd codon positions of the 13 PCGs sequences (Figure S2b). Among them, the topology of phylogenetic tree based on whole genome sequences has the highest support. The results of the phylogenetic tree indicate that *T. canaliculata* and *Issikiopteryx taipingensis* have the closest genetic relationship with a bootstrap value of 100 (Figure 3). *T. canaliculata* belongs to the subfamily Torodorinae, *I. taipingensis* belongs to the subfamily Lecithocerinae, so these two species belong to the same family of Lecithoceridae.

## Discussion and conclusion

In this study, the complete mitochondrial genome of *T. canaliculata* was first reported, and conforms to the typical lepidoptera arrangement in base composition (T > A > C > G) and gene arrangement with the gene order *trnM*-*trnI*-*trnQ* at the same junction (Jeong et al. 2022). Compared to the phylogenetic tree of the superfamily Gelechioidea constructed by nuclear genes (Wang and Li 2020), the phylogenetic tree constructed based on the 13 PCGs does not well support the interfamily phylogenetic relationships of the Gelechioidea, as the bootstrap value between different families in the nuclear gene tree is significantly higher. Nevertheless, the mitochondrial genome with different genetic source from nuclear genes can still provide a new genetic perspective for the phylogeny of the Gelechioidea. In summary, the results of this study have added molecular data to the understanding of the phylogenetic position of the Lecithoceridae, and provided more information on the evolutionary relationship of the Gelechioidea.

## Supplementary Material

Figure S2.jpg

Figure S1.png

## Data Availability

The genome sequence data that support the findings of this study are openly available in GenBank of NCBI at [https://www.ncbi.nlm.nih.gov/nuccore/PP187732.1] under the accession no. PP187732. The associated BioProject, SRA, and BioSample numbers are PRJNA1075444, SRR28369309, and SAMN40506120, respectively.
