# Supplementary figures and images for: The complete mitogenome of Torodora canaliculata (Yu & Wang, 2022) (Lepidoptera: Lecithoceridae) and its phylogenetic implications
Source: Mitochondrial DNA B Resour. 2025 Feb 20;10(3):248–52. doi: 10.1080/23802359.2025.2468752 (PMC11843649; doi:10.1080/23802359.2025.2468752)

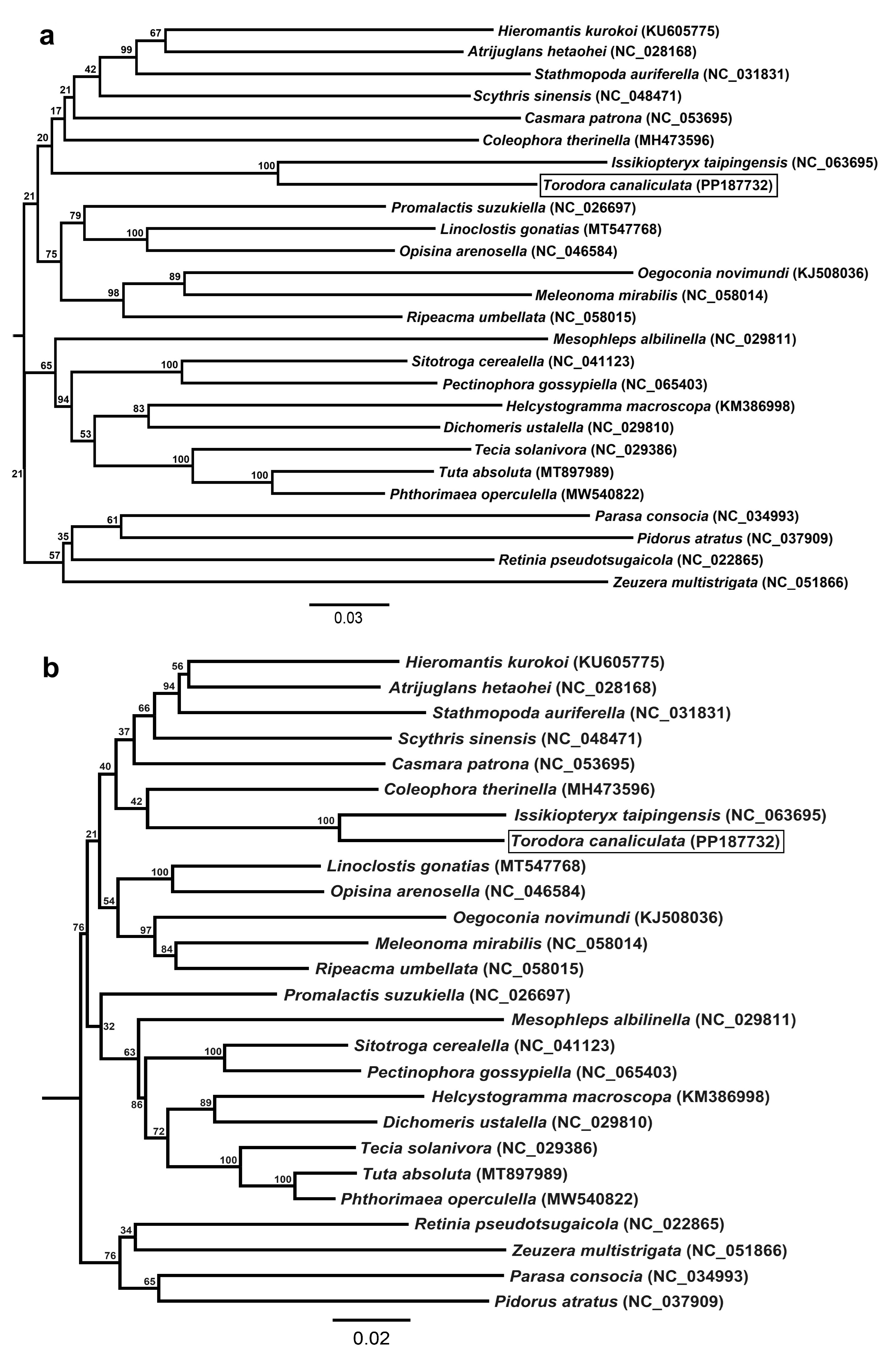

Supplement: Figure S2.jpg [file TMDN_A_2468752_SM6487.jpg]

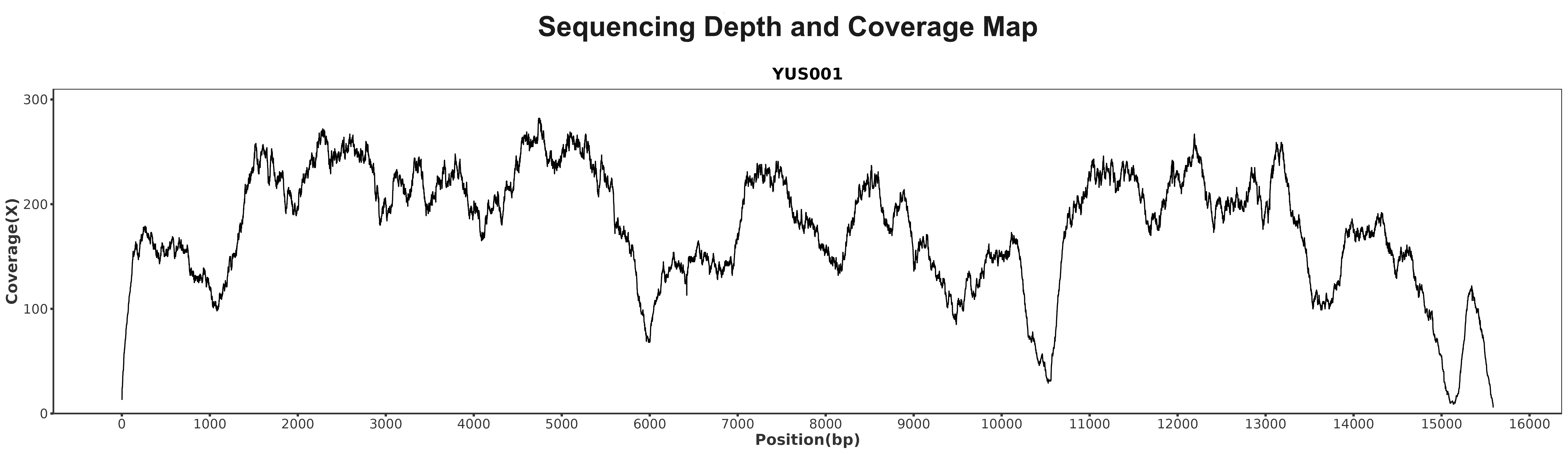

Supplement: Figure S1.png [file TMDN_A_2468752_SM6486.png]
